# Self-care towards the end of life: A systematic review and narrative synthesis on access, quality and cost

**DOI:** 10.1177/02692163241286110

**Published:** 2024-10-19

**Authors:** Joshua Gallagher, Bárbara Antunes, James Sutton, Isla Kuhn, Michael P Kelly, Robbie Duschinsky, Stephen Barclay

**Affiliations:** 1 Primary Care Unit, Department of Public Health & Primary Care, University of Cambridge, Cambridge, UK; 2 Cambridge University Medical Library, Cambridge, UK

**Keywords:** Self-care, self-management, self-monitoring, end of life, palliative care, digital health, medical technology, cost, cost-effectiveness, quality of life

## Abstract

**Background:**

Policy and practice encourages patients to engage in self-care, with individual patient management and remote monitoring of disease. Much is known of the moderate stage of chronic disease, without a clear understanding of how self-care applies towards the end of life.

**Aim:**

To review the current evidence on practices of self-care in life-limiting conditions and its impacts on healthcare utilisation, quality of life and associated costs.

**Design:**

We systematically searched 10 scientific databases (MEDLINE, CINAHL, Embase, PsycINFO, Cochrane Central, Cochrane Database of Systematic Reviews, Scopus, Sociological Abstracts, Social Work Abstracts and Health Management Information Consortium) from inception to October 2023 with citation and hand searching. A narrative synthesis was undertaken, with quality and relevance assessed using Gough’s Weight of Evidence framework. Titles and abstracts were independently screened by three researchers.

**Results:**

Findings from 33 studies revealed self-care as increasingly burdensome or unfeasible towards the end of life, with patients delaying use of professional care. Self-care became increasingly burdensome for patients, carers and professionals as illness progressed. Self-monitoring may exacerbate hospitalisations as patients delayed seeking professional help until crises arose. Findings regarding quality were inconclusive, with some evidence suggesting that self-care can decrease care costs.

**Conclusions:**

This review has shown that research on self-care is an evolving area of study, with a current focus on acute care and hospitalisations. Future research should seek to provide a more complete account of the relation between self-care and non-acute care use, and quality, with further efforts to study self-care costs incurred through self-funding.

**What is already known about the topic?**

1. Self-care is an increasingly advocated aspect of chronic disease management.
2. In chronic disease, self-care can lower care utilisation and health system cost.

**What this paper adds?**

1. Self-care has potentially negative consequences for patients as they approach death.
2. Although self-care can be cost-effective in acute care contexts, the benefit on service use and quality is not clear.
3. Current evidence on self-care is focussed on early to moderate stages of disease and on a limited number of institutional settings.

**Implications for practice, theory or policy**

1. Practitioners need to take caution when encouraging self-care as illness progresses, as its efficacy weakens as the end of life approaches.
2. Future self-care research is needed for patients in community and non-acute care settings, with attention to self-funding of care.

## Background

Current and expected rises in the proportion of people living with debilitating and life-limiting conditions poses a challenge to how health and social care are provided.^1^ ‘Self-care’ forms a part of symptom management strategies^2^ and is used to encompass a range of activities carried out by an individual to maintain their health through adherence to treatment, monitor their condition and their efforts to manage the effects of illness independently. These are respectively termed ‘self-care maintenance’, ‘self-care monitoring’ and ‘self-care management’.^3–6^

The relationship between the concepts of self-care and ‘self-management’ is unclear^7–9^ with a multitude of, often conflicting, terms used to describe both terms with no univocal definition of the former.^10^ Seminal work^11^ in chronic illness outlines three relational processes involved in self-care: (1) maintenance (defined as adherence to medical regimes and treatments), (2) monitoring (objective measurements and subjective experiences) and (3) management (non-medical strategies used by patients). Other authors have found self-management to be a distinct but related process that operates within self-care.^4,5,7,12^

Self-management is well-established in chronic illness research^5^ and has been highlighted as an important future direction for palliative care practice and research.^13^

Much research in self-care at the end of life has investigated the ‘support’ of self-management tasks at the periphery of self-care^14,15^: lacking accounts of how patients themselves engage in these activities. Self-management support is defined as the ‘*assessing, planning, and implementing appropriate care to enable the patient to live until they die and supporting the patient to be given the means to master or deal with their illness or their effects of their illness themselves’*.^16^ Others have examined patient, carer and professionals’ perspectives on how best to support patients from the stance of appropriate professional roles.^17^ It has been advocated instead that a decentring exercise of self-care is needed, away from professionals and ensuring that strategies are ‘owned and used by people who are ill’ to ensure that patients are in charge of their own care.^13^

Self-care has an increasingly important role in how people live and die. Advances in digital health and technology have meant that some services traditionally provided in hospitals and clinics can now take place in patients’ own homes, posing new challenges for how self-care can be carried out in the community.^18^ The recent COVID-19 pandemic accelerated this shift towards the in-home management and remote monitoring of disease.

It has been noted that self-care in the end of life context is under-researched.^19^ Studies on the effects of self-care are often limited to the early to moderate stages of chronic disease,^19–22^ where self-care can increase peoples’ feelings of control, reduce care use and lowers the overall cost for health and social care systems.^23–25^ The limited research on the end of life self-care has most often been conducted with cancer patients, or centred on the role of informal carers.^15^ The later stages of chronic disease, towards the end of life, pose numerous challenges for patients, families and health and social care systems, including maintaining quality of life and minimising care costs.^25^ As patients become increasingly unwell and less able to look after themselves, the role and efficacy of self-care shifts and is brought into question. Understanding perceptions of self-care and whether it can improve patient outcomes at this difficult time is thus of paramount importance.

### Objectives

To review the current evidence on practices of self-care in life-limiting conditions and its impacts on healthcare utilisation, quality of life and associated costs.

We examine the current empirical evidence on self-care with a focus on palliative and end of life care populations. Regarding self-care for adults at home towards the end of life, we seek to answer the following questions:

1. What are patient, family and professional views of self-care?
2. How does self-care affect formal care utilisation?
3. What is the impact of self-care on patients’ quality of life?
4. How does self-care affect overall cost at the end of life (for patients, families and services)?

## Methods

### Design

A narrative synthesis approach was used to integrate findings from a range of study types.^26^ We deemed this chosen method as the most exhaustive approach to analysing findings from studies that used qualitative, quantitative and mixed-methods designs. The review questions speak to a mixture of qualitative (views) and quantitative (use, quality and cost) studies. Study quality and relevance was assessed using Gough’s Weight of Evidence framework.^27^ Our initial research protocol was registered with PROSPERO (CRD42021242259) and served as the basis for our review. The design of the current review differs in scope from our original protocol.

### Search

Following an initial scoping search on self-care, inclusion and exclusion criteria were clarified and search strategies developed in discussion with the review team’s Information Scientist (IK). Ten databases were searched from October 2023 to inception: MEDLINE (Ovid), CINAHL (Ebsco), Embase (Ovid), PsycINFO (EBSCO), Cochrane (Central, Database of Systematic Reviews), Scopus and Sociological Abstracts (ProQuest), Social Work Abstracts (ProQuest), and Health Management Information Consortium (OVID). Additional papers were sought through a hand-search of Palliative Medicine journal from January 2010 to October 2023 with reference and citation searches of all included papers through Google Scholar. Search terms (Table 1) were developed from a scoping search of the literature, health and social care related glossaries^28–32^ and consultations with a medical librarian (IK).

**Table 1.** Example search strategy (Medline).

Epub Ahead of Print, In-Process and Other Non-Indexed Citations, Ovid MEDLINE(R) Daily and Ovid MEDLINE(R) 1946 to Present
exp ‘self-management’ / or exp ‘self care’ / or exp ‘self administration’ / or exp ‘self efficacy’ / or exp ‘self-assessment’ / or exp ‘self-control’ / or exp ‘self medication’ / or exp ‘self-testing’ / or exp ‘diagnostic self evaluation’ / or exp ‘self-help devices’ / or exp ‘self-neglect’ / or exp ‘patient generated health data’ / or exp ‘blood pressure monitoring, ambulatory’ /
OR
(Self car* or self monitor* or self help or self medicat* or self treat* or self alleviat* or self efficac* or self govern* or self regulat* or self evaluat* or self measur* or self maint* or self us* or self educat* or self test* or self diagnos* or self refer* or self manag* or self examin* or self inject* or self admin* or self admit* or self admis* or self support* or self diagnos* or self collect* or self screen* or self surveil* or self sampl* or self exam* or self triag* or self heal* or self assess* or patient activ* or self rehab* or self nurs* or self feed* or self nour* or self drink* or self hydrat* or self clean* or self wash* or self bath* or self groom* or self catheter* or self observ* or self prepar* or self intub* or self dress* or self extub* or self insert* or self appl* or self transport* or self irrigat* or self financ* or self instill* or self fund* or self transfer* or self therap* or self audit* or self recover* or self discharg* or self experiment* or self restrict* or self restrain* or self mediat* or self provi* or self prevent* or self procur* or self cop* or self prognos* or self isolat* or self prescri* or self inform* or self relie* or self induc* or self aware*).ti,ab.
AND
exp Terminal Care/ or exp Palliative Care/ or exp ‘Hospice and Palliative Care Nursing’/ or exp death/ or exp Palliative Medicine/ or exp Terminally Ill/ or ((end adj2 life) or ((final* or last*) adj1 (hour* or day* or minute* or week* or month* or moment*)) or palliat* or terminal* or (end adj stage) or dying or (body adj2 (shutdown or shut* down or deteriorat*)) or deathbed or incurable or hospice* or (late adj stage*)).ti,ab.
3853 results (with limits: human, English language)

### Paper selection and extraction

**Figure 1.**
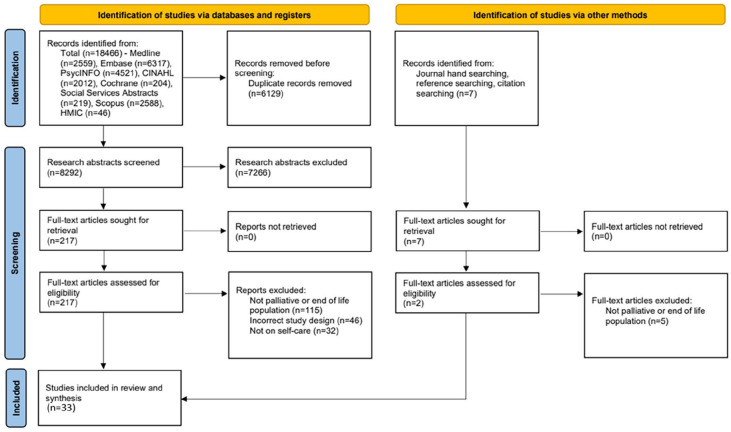
PRISMA flow chart of search strategy.

Our search (Figure 1) began by excluding duplicate records.^33^ Titles and abstracts were then independently screened by JG, BA and JS, with subsequent screening of full text papers in-line with inclusion and exclusion criteria (Table 2). Operationalising the term ‘end of life’ is not an easy task; the imprecision of when a person is defined as being at the end of life is widely acknowledged. Our definition of the ‘end of life’ draws on previous work in advanced disease.^34,35^

**Table 2.** Eligibility criteria.

Concept	Inclusion criteria	Exclusion criteria
Self-care	Patient initiated activities relevant to the maintenance, monitoring and management and of disease.	Self-care activities without a significant proportion of patient initiation.
Patient population	Findings concerning adults with at least two of the following three criteria: 1. Advanced disease: (a) a specified, palliative chronic illness included in the ICD10 code filter,^36^ (b) multimorbidity or frailty, (c) an unplanned hospital admission in the past year; 2. Identified as being part of a ‘palliative care’ population; 3. Identified as being in their last year of life.	People under 18 years, those unlikely as being towards the end of life.
Setting	People living in their home residences.	People resident in health or social care facilities (including in-patients).
Study characteristics	Peer-reviewed empirical research which features data pertaining to the review objectives relevant to patients, their family members or the professionals in charge of their care.	Reviews or meta-analyses with no new empirical data, editorials, opinion pieces, individual case studies, conference abstracts or research protocols.
Language	Studies with findings presented in the English language.	Studies not in the English language.

Data from full text articles were extracted into a review-specific data extraction form. JG, BA and JS appraised the quality and relevance of each included study using Gough’s^27^ ‘Weight of Evidence’ framework which allows for assessments to be made of both quantitative and qualitative studies. Any discrepancies in quality appraisals were first discussed between JG, BA and JS, with subsequent approval sought from SB if an agreement between JG, BA and JS could not be achieved, to reach consensus.

### Data synthesis

The review design embodied a narrative approach, as described by Popay et al.^26^ to integrate findings from a range of study types; quantitative, qualitative and mixed-methods. Narrative syntheses take a textual approach to data synthesis to ‘tell the story’ of an underlying phenomenon. In line with Popay’s approach to narrative synthesis we undertook a preliminary synthesis which involved data extraction of results from each paper which met the inclusion criteria. This data extraction took place in three stages. Studies were first individually analysed by creating a textual description using a review-specific data extraction form. These descriptions were then tabulated according to the review questions they pertained to.

We then explored relationships in the data, with a focus on explaining any differences in the direction and size of the effects identified. Following data extraction and tabulation, thematic analysis was carried out by JG to identify the main, recurrent and corresponding data across included studies. Thematic analysis brought together data from included studies to answer each review question. Differences in research methods or orientations across studies were discussed between JG and SB and a strategy for synthesis was agreed. Heterogeneity among studies was explored by JG and BA through cross-tabulation of themes and study findings which were discussed between JG and BA for consensus with additional input from clinicians, patients in patient and public involvement sessions and the review team (SB, MK and RD).

The narrative synthesis concluded with an assessment of robustness of the synthesis using Gough’s Weight of Evidence Framework. Studies were assessed by JG, JS and SB and weighted by their ranking as high, medium or low weight of evidence. Studies judged as having a low Weight of Evidence were not included in the synthesis unless they presented findings corresponding to evidence from medium or high Weight of Evidence studies.

## Findings

33 studies were included (Figure 1) (Table 3) from UK (*n* = 6), USA (*n* = 5), Netherlands (*n* = 7), Sweden (*n* = 3), New Zealand (*n* = 1), Australia (*n* = 1), Uganda (*n* = 1), Norway (*n* = 2), Japan (*n* = 1), Italy (*n* = 2), Brazil (*n* = 2), Ireland and Belgium (*n* = 1) and Germany (*n* = 1). Eligible studies encompassed findings from 2882 patients, 7 informal caregivers and 221 professionals and included a meta-study which did not disclose the total population reviewed. Study designs were qualitative (*n* = 17), quantitative (*n* = 11) and mixed-methods (*n* = 5), including 6 randomised-control trials. Seventeen studies presented data on self-care monitoring, 14 on self-care maintenance and 11 on self-care management. Data were presented on views of self-care (*n* = 19), care utilisation (*n* = 7), quality (*n* = 5) and cost of self-care (*n* = 3). Weight of Evidence was high (*n* = 9), medium (*n* = 19) and low (*n* = 5). Table 3 presents findings from the included studies.

**Table 3.** Studies included in the review and synthesis (*n* = 33).

Reference	Research aim(s)	Research methods	Participants	Self-care aspect	Relevant finding(s)	Weight of evidence	Key findings
Cleland et al.,^37^ USA.	To identify whether home telemonitoring improves outcomes compared with nurse telephone support and usual care for patients with heart failure who are at high risk of hospitalisation or death.	Quantitative, RCT, intention-to-treat analysis and Cox regression	426 persons with heart failure	Monitoring	Utilisation	High	Home self-monitoring was associated with more hospital admissions but a significant reduction in the average duration of hospital stay when compared to a control group receiving nurse telephone support (10.9 days vs 14.8 days, respectively).
Sand et al.,^38^ Norway.	To explore patients’ experiences of using medicines when they are living with far-advanced cancer and short life expectancy.	Qualitative, semi-structured interviews, thematic analysis	15 persons with cancer	Maintenance	Patient views	High	Self-management, not compliance, was a key issue for patients. Patients self-regulated their own prescribed dosages and were ambivalent about taking medication which they knew they needed but did not want to take due to a fear of dying in a state of addiction. This was based on patients family histories. Patients’ self-regulation was often at odds with treatments that doctors’ prescribed.
Munck et al.,^39^ Sweden.	To describe district nurses’ conceptions of medical technology in palliative home care.	Qualitative, semi-structured interviews, phenomenographic analysis	16 district nurses	Monitoring	Professional views	Medium	District nurses felt more knowledge of patients was expected of them with self-monitoring technology in place. Self-monitoring allowed palliative patients to maintain their independence and autonomy. Having self-monitoring technology in the home was viewed as giving patients an assurance that they will die at home.
Cox et al.,^40^ UK.	To evaluate the support provided to persons living with lung cancer using a computerised assessment tool and to determine the clinical acceptability of the tool in a palliative care setting.	Qualitative, semi-structured interviews, framework analysis	13 palliative care clinicians	Monitoring	Professional views	Low	Clinicians felt that highly anxious patients could cause unnecessary alarm through monitoring. They were also concerned that it could exclude face-to-face support for patients and eliminate the clinician’s opportunity to visually assess the patient and use their clinical judgement.
Enguidanos,^41^ USA.	To determine the perspectives of seriously ill individuals on reasons for 30-day hospital readmission.	Qualitative, semi-structured interviews, thematic analysis	9 persons with heart failure	Management	Patient views/utilisation	Medium	Patients described how they had too much to manage on their own without extra support. Failure to self-care and manage their health conditions adequately resulted in more health problems and complications which led to hospital admission.
Buck et al.,^42^ Italy.	To understand the contribution of comorbidity to heart failure self-care behaviours and outcomes and whether comorbidity is a moderator of the relationship between self-efficacy and heart failure self-care behaviours.	Quantitative, cross-sectional, structural equation model and slope analysis	628 persons with heart failure	Maintenance/Management	Utilisation/Quality	Medium	Higher self-care maintenance (Self-Care of Heart Failure Index) was associated with higher physical and emotional quality of life (Minnesota Living with Heart Failure Questionnaire) and fewer hospitalisations; higher self-care management was associated with lower emotional quality of life.
Dong et al.,^43^ Australia.	To describe the beliefs, attitudes and experiences of patients with multiple symptoms in advanced cancer.	Qualitative, semi-structured interviews, thematic analysis	35 persons with cancer	Monitoring/Maintenance	Patient views	High	The responsibility of self-management, perpetual monitoring and quantifying subjective symptoms, was seen as burdensome and anxiety-provoking. Many feared they would incorrectly report symptom intensity or forget to bring up recent symptoms. Many patients accepted, took pride in and gained control by ‘tweaking’ their medication.
Hughes et al.,^19^ UK.	To ascertain the views of specialist palliative care professionals on patient self-management of cancer pain.	Qualitative, focus group interviews, content analysis	6 community nurses, 3 CTs, 5 hospice nurses, 1 hospice SW, 1 hospice spiritual carer, 1 palliative care consultant	Maintenance/Management	Professional views	Medium	Clinicians felt that self-management gave patients a measure of control over what is happening to them. However, at a late stage of their disease, patients were too fatigued to manage their conditions. Their concentration was quite poor, with multiple symptom burden leaving them unable to have the energy to do things on their own.
Karasouli et al.,^44^ UK.	To explore patient and carer experiences and those of their healthcare professionals in the period leading up to emergency admission to hospital.	Qualitative, semi-structured interviews, cross-case and thematic analysis	24 persons with cancer, 15 persons with COPD	Management	Utilisation	Medium	There were three phases which led to an emergency admission: self-management as the condition deteriorated, negotiated decision-making and letting go. Patients tended to ‘hold on’ by monitoring their symptoms and self-medicating to alleviate them. Patients eventually reached a threshold where emergency admission was seen as the only viable option.
Bennet et al.,^45^ UK.	To develop a self-management support toolkit and delivery strategy, and to test the feasibility of evaluating this intervention in a future definitive trial.	Mixed-methods, surveys and semi-structured interviews, cost-effectiveness and thematic analysis	15 persons with cancer	Maintenance	Cost	High	Cost–utility analysis suggested that the intervention appears to be cost-effective compared with standard care alone (0.0045 QALYs per patient) and could lead to cost savings.
Namukwaya et al.,^46^ Uganda.	To explore the beliefs of patients with heart failure, their understanding of their illness and its treatment and how this influenced their health-related behaviour.	Qualitative, in-depth interviews, grounded theory	21 persons with heart failure	Maintenance/Management	Patient views	Medium	Many patients engaged in their own management strategies to supplement medical treatment. Participants’ perceptions of self-care reflected the contemporary lay views on what good health is and how to achieve it. Self-care allowed some participants to avoid dependence and feel more responsible for their health.
Slev et al.,^47^ Netherlands.	To gain more insight into how nurses perceive their role in self-management support for people confronted with advanced cancer and their opinions about the use of eHealth in this regard.	Qualitative, focus group interviews, thematic analysis	45 cancer nurses	Monitoring	Professional views	Medium	Nurses said that eHealth can let patients remain in control, by allowing patients to ask a healthcare professional questions and enables online contact with peers. Some hospice nurses saw the main potential added value of eHealth in the care of patients in the early palliative phase. Patients often no longer have enough energy to use a laptop or tablet, for example, in the final phase.
Lee et al.,^48^ Italy.	To identify patterns of self-care behaviours in patients with heart failure and their associations with clinical events.	Quantitative, retrospective cohort, latent class analysis	459 persons with heart failure	Maintenance/Management	Utilisation	Medium	Patients who engaged in self-care maintenance and management (Self-Care of Heart Failure Index) were less likely to require hospitalisation than those who did neither (Adjusted Hazard Ratio = 0.664). Patients who focussed only on maintenance were as likely to be hospitalised as patients who didn’t manage or maintain. Patients with poor self-care behaviours may utilise less healthcare resources in the short term, but ultimately poor self-care appears to result in worse clinical event-risk.
Meads et al.,^49^ UK.	To conduct a case study economic evaluation of two pain self-management interventions compared with usual care.	Quantitative, meta-review, cost-effectiveness analysis	17 reviews of persons with cancer*	Monitoring	Cost	High	Two self-care monitoring interventions (PainCheck and the Tackling Cancer Pain Toolkit) were both found to be cost-saving and more effective (QALYs) than usual care.
Gilbertson-White et al.,^50^ USA.	To engage stakeholders about the symptom management needs and concerns of patients with advanced cancer living in rural areas.	Mixed-methods, surveys and semi-structured interviews, descriptive statistics and thematic analysis	16 persons with cancer, 10 clinicians	Monitoring	Patient, Professional views	Medium	Some patients in the early stages of disease were able to self-manage without much difficulty while other patients with many concurrent symptoms became too overwhelmed.
Greer et al.,^51^ USA.	To test the efficacy of a tailored cognitive-behavioural therapy mobile application to treat anxiety in patients with incurable cancer.	Quantitative, RCT, analysis of covariance	145 persons with cancer	Maintenance	Quality	Medium	No significant differences were seen in quality of life (Functional Assessment of Cancer Therapy-General) between intervention and non-intervention groups.
Matsuda et al.,^52^ Japan.	To examine the effect of a self-monitoring quality of life intervention on global quality of life, and physical and emotional function in patients with cancer receiving palliative care.	Quantitative, RCT, intention-to-treat analysis and linear mixed-effects model	43 persons with cancer	Monitoring	Quality	Medium	The self-monitoring quality of life intervention (Care Notebook) showed improvement in global quality of life (EORTC QLQ-C-15-PAL) and physical function among patients in the study.
Freilich et al.,^53^ Sweden.	To explore professionals’, patients’ and family caregivers’ perspectives on how primary healthcare professionals should support self-management in patients with multimorbidity.	Qualitative, focus group interviews, pair interviews, individual interviews, content analysis	12 persons living with multimorbidity, 20 physicians, 3 registered nurses, 7 family caregivers	Monitoring/Maintenance	Patient, Family, Professional views	Medium	Professionals described how patients who managed their diseases independently gave them valuable time to see other patients. For most patients and family caregivers, recording information felt meaningful and became an important daily routine. For others, this shift in responsibility felt challenging at times, and when patients became unwell they were unable to self-monitor.
Helleman et al.,^54^ Netherlands.	To evaluate the use of telehealth as part of specialised care for patients with amyotrophic lateral sclerosis and the user experiences of patients and healthcare professionals.	Mixed-methods, surveys and semi-structured interviews, descriptive statistics and thematic analysis	23 persons with motor neurone disease, 9 healthcare professionals	Monitoring	Patient, Professional views	Medium	The most frequently reported reason for discontinuing self-monitoring was that patients felt there was no added benefit due to the end-of-life phase or death (*n* = 12). Enabling factors for self-monitoring use were low self-monitoring burden, user-friendly platforms and the provision of personalised feedback.
Radionova et al.,^55^ Germany.	To explore occupational routines, attitudes and expectations of physicians and nurses with regards to a planned electronic assessment system of patient reported outcomes.	Qualitative, semi-structured interviews, content analysis	1 hospice physician, 3 hospice nurses, 5 hospital physicians, 3 hospital nurses, 4 GPs, 1 district nurse, 2 specialist nurses	Monitoring	Professional views	Medium	Clinicians viewed standardised patient reported outcomes as not adequately reflecting the subjective reality of patients and the complexity of their needs. They further questioned the ability of patients to self-assess their conditions and well-being and thereby the doubted validity of the patient reported outcomes.
de Veer et al.,^56^ Netherlands.	To gain an understanding of the perceptions of persons with incurable cancer regarding a nurse-led self-management support intervention with an integrated eHealth application and its potential effectiveness.	Mixed-methods, surveys and semi-structured interviews, paired t-test and thematic analysis	36 persons with cancer	Monitoring	Quality	Medium	No statistically significant differences were observed in overall quality of life (EORTC-QLQ-C15-PAL) between pre-intervention and post-intervention results.
Francis et al.,^57^ New Zealand.	To explore how people with complex, established co-morbidities experience long-term condition care.	Qualitative, multiple case studies, thematic analysis	16 persons living with multimorbidity, 16 GPs, 8 nurses	Maintenance	Patient, Professional views	Low	Patients experienced multiple losses in function leaving them with insufficient energy required to self-manage and feeling disempowered. Self-management was at odds with advanced disease and increased patients’ sense of personal failure. Clinicians felt unable to offer care that would benefit patients from the inflexible restrictions of the self-management approach.
Alvariza et al.,^58^ Sweden.	To explore palliative care nurses’ work experiences caring for patients at the end of life in private homes.	Qualitative, photo-elicitation, interpretive description	10 palliative care nurses	Management	Professional views	Low	Nurses were concerned that self-care could be at odds with safe care. Nurses had to trust in patients’ and family members’ abilities to perform self-care and comply with advice. A balance of self-care and safe care was achieved through planning of care activities in collaboration with patients and family members.
Mahl et al.,^59^ Brazil.	To evaluate the delay in care for persons living with cancer in post-treatment follow-up or palliative care during the COVID-19 pandemic, and its impact on health outcomes.	Quantitative, Mann-Whitney U test and Fisher exact test	31 persons with cancer	Maintenance	Utilisation	Low	There was an association between delayed cancer care and the use of self-medication (self-reported). Patients reporting delays in cancer care during the COVID-19 outbreak were more likely to use self-medication with a risk of duplicate prescribing, medication overdoses and adverse interactions.
Doyle et al.,^60^ Ireland/Belgium.	To design and develop a digital health platform for facilitating older adults self-managing multimorbidity, with support from their care network and evaluate end user engagement and experiences with this platform.	Mixed-methods, surveys and semi-structured interviews, descriptive statistics and thematic analysis	120 older persons living with multimorbidity	Monitoring	Patient views	Medium	Self-monitoring was viewed by patients as giving them a sense of control in patient-doctor interactions. Patients distrusted their own self-monitoring readings which led to disengagement. For some, monitoring with a device felt like an added burden. Three participants in Ireland withdrew from the study as they had enough to deal with in managing their conditions on top of self-monitoring
Oelschlägelet al.,^61^ Norway.	To explore healthcare professionals’ experiences regarding the significant challenges, facilitators and assessments associated with implementing a technological solution in palliative home care for patients with cancer.	Qualitative, focus group interviews and semi-structured interviews, content analysis	2 specialised cancer nurses, 2 nurses, 1 SW, 1 PTs, 2 OTs	Monitoring	Professional views	Medium	Self-monitoring as patients’ condition declined was a reminder for some of impending death. According to professionals, some patients were unable to use technology. Nurses viewed monitoring as having increased possibilities for professionals to help and to reach more patients.
Csipke et al.,^62^ UK.	To investigate a social intervention to promote living well and enhance independence for people with mild dementia.	Quantitative, RCT, descriptive statistics	73 persons with dementia	Management	Quality	Medium	No significant differences in quality of life (DemQol) were found between the intervention group and usual care group.
Noorlandt et al.,^18^ Netherlands.	To obtain insight in self-management challenges of persons with advanced cancer and factors that influence their self-management.	Qualitative, in-depth interviews, inductive thematic analysis	33 persons with cancer	Management	Patient views	High	Self-care gave patients a feeling to be ‘in charge’ of their health. Most participants reported wanting to live ‘as normally as possible’, and their self-management sometimes threatened this goal. Some participants described their self-management as being ‘normal’ to them and were not consciously aware of their practices.
van Dongen et al.,^63^ Netherlands.	To examine healthcare professionals’ views on self-management and self-management support.	Qualitative, semi-structured interviews, thematic analysis	6 cancer medical specialists, 6 nurses specialists, 8 GPs, 7 home care/hospice nurses	Monitoring/Maintenance	Professional views	High	Most professionals felt self-management in advanced cancer made their jobs ‘bigger’ and ‘more varied’. Professionals had to keep up to date on their patients self-monitoring, and wider lifestyle trends used by patients to self-manage. Self-management was especially challenging when there were differences between patient and professional circumstances, capabilities, opinions on viable strategies and responsibilities.
Schuit et al.,^64^ Netherlands.	To determine the efficacy of a self-monitoring intervention compared to care as usual among incurably ill patients living with cancer with a life expectancy of more than three months.	Quantitative, RCT, intention-to-treat analysis and linear mixed-effects model	138 persons with cancer	Monitoring	Quality	High	Health related quality of life (EORTC-QLQ-C15-PAL) did not differ significantly between the self-monitoring intervention group and control group.
Schuit et al.,^65^ Netherlands.	To assess the cost-utility of the eHealth application among incurably ill persons with cancer, compared to care as usual.	Quantitative, RCT, cost-effectiveness analysis	138 persons with cancer	Monitoring	Cost	High	Among patients with incurable cancer, the self-monitoring intervention did not impact costs and was slightly less effective compared to usual care (−0.02 and −0.01 QALYs, respectively).
Noguez et al.,^66^ Brazil.	To analyse the self-care of cancer ill people at the end of their lives; patients staying under Palliative Care in a home care service.	Qualitative, unstructured observations, narrative interviews, narrative analysis	11 persons with cancer	Maintenance/Management	Patient views	Low	Patients used a combination of biomedical, traditional medical and spiritual modalities of self-care. Narratives showed that to manage biomedical self-care, participants decided and chose not to follow the physician’s prescription regarding the use of certain medications. Out-of-pocket expenses for treatments that didn’t work were a concern.
Freedland et al.,^67^ USA.	To test the hypotheses that better heart failure self-care is associated with a lower rate of all-cause readmissions, and that readmissions motivate patients to improve their self-care.	Quantitative, prospective cohort, linear mixed-effects linear model	400 persons with heart failure	Maintenance/Management	Utilisation	Medium	Higher Self-Care of Heart Failure Index Maintenance scores predicted more rather than fewer readmissions. More readmissions likewise predicted higher Maintenance scores.

RCT: randomised-control trial; COPD: chronic obstructive pulmonary disease; GP: general practitioner; PT: physio-therapist; OT: occupations therapist; CT: complementary therapist; SW: social worker.

* Total number of participants not disclosed in reviews.

### Views of patients, professionals and caregivers

The most prevalent theme in studies of patient and professional views was the burden placed on participants in monitoring^50,58^ and managing their own conditions.^41^ Most notably, as patients approached the end of life, self-care monitoring^43,47,54,58^ and maintenance^19,57^ became increasingly unfeasible due to illness and symptom burden, which, for some patients, could lead to a sense of failure,^57^ due to the ‘goal oriented’ nature of the self-management approach. Patients^43^ and professionals^61^ remarked that self-care monitoring across the dying trajectory could, in some cases, remind patients of their impending death as they viewed their decline over time. Some professionals did consider that that self-monitoring technology could give an assurance to patients that they would die at home.^39^ As death approached, self-care could give patients a feeling of being ‘in charge’ of their health.^39^

In the earlier stages of illness, patients and clinicians viewed self-care positively, giving patients a feeling of responsibility and independence.^39,60^ Patients felt able to exercise this independence, at times by adjusting their own medications,^18,39,58,60^ at times fuelled by a fear that they would end their lives addicted to prescribed analgesics.^38,46^ In one study, as the level of symptom burden increased, patients were described by clinicians as having a tendency to engage in their own alternative, and sometimes extreme, self-care management strategies which could compromise their safety and conflict with formal care provision, particularly in the case of self-medication.^57,63^

This was also seen in patients’ own accounts of how they managed their conditions using unconventional methods.^18,46^ In one study, participants reported that self-care management sometimes threatened their ability to live ‘as normally as possible’, despite their self-management as also being perceived as part of a background ‘normal’ practice.^18^ Though desiring to help patients with their illnesses, clinicians felt hindered in providing care as they did not know the full picture of patients’ self-care management which may negatively affect their prescribed treatments.^38^ Nurses too felt that what patients understood as ‘self-care’ could be opposed to ‘safe care’, with the clinicians’ job seen as being one of ensuring a balance between self-care and safe care.^28^

The ability of patients to accurately self-assess their symptoms and well-being was a concern for patients^58,60^ and healthcare professionals.^40,55,60^ Some patients felt that placing an objective value on their subjective well-being was a challenge and led to a worry that incorrect reporting may result in unnecessary concern for their healthcare providers.^43^ Using self-monitoring, patients felt empowered as they had more data on their conditions to present to healthcare professionals during consultations.^60^ This view was echoed in an interview study with cancer nurses.^47^ Doctors mentioned that they were able to see more patients with the time saved through patients self-monitoring their own conditions.^53^ Similarly for self-care management, some clinicians commented that it gave them more time for other patients but at the same time had made their work ‘bigger and more varied’ as they had to spend time learning about new management techniques patients were using to cope with their symptoms.^63^ This was similarly seen in self-monitoring as healthcare providers felt an expectation to keep up-to-date on all of the new information patients had generated through monitoring.^40,63^ and overall tended to mistrust patient measurements of symptom burden and well-being particularly when concerning psychological well-being,^43,50^ favouring in-person consultations and clinical assessments.^40^

### Use of care towards the end of life

Seven studies presented data pertaining to self-care and care use towards the end of life.^37,41,42,44,48,59,67^ The evidence concerning effect of self-care maintenance on hospital admissions was contradictory, either increasing^67^ and decreasing^42^ or having no effect.^48^ Similarly self-care monitoring was associated with more hospitalisations^37^ or decreased admissions when combined with maintenance behaviours.^42^

Among patients with heart failure, a latent class analysis of 459 patient cases, found that patients who engaged in a combination of self-care maintenance and management had a lower number of admissions to an acute care facility.^48^ Yet, for patients who were identified as self-care ‘maintenance focussed’ levels of hospitalisation did not differ from those who were neither effective at both self-care management and self-care maintenance. On the contrary, one study found that higher self-care maintenance was associated with fewer hospitalisations; whereas self-care management was not associated with hospitalisation.^42^ In a study of 400 persons with heart failure in the USA,^48^ higher maintenance was longitudinally associated with a higher number of readmissions to hospital, with management not being a significant predictor of hospital admission in their model.

Among a low-quality sample of persons with cancer, a bivariate association was found from a study between self-care maintenance and delayed access to cancer care, though the direction of the relationship was not made clear, with the lack of access to formal care (due to the COVID-19 pandemic) acting as a potential reason for engaging in self-care.^59^

An RCT of 426 patients with heart failure of a home self-monitoring intervention, compared with nurse telephone support, found those receiving the intervention were more likely to be admitted to hospital, though the mean duration of admissions was reduced (10.9 days vs 14.8 days, respectively which they perceived as due to higher confidence of clinicians to discharge patients home with the support of patient self-monitoring.^37^

Qualitative studies described how self-care maintenance was used by patients a means of avoiding hospital admission.^41,44^ In the case of emergency admission in heart failure, patients often carried out self-care to avoid hospital admission for as long as possible, despite their deterioration and were actively ‘holding on’ through monitoring their symptoms and self-administering medication.^44^ This resistance to assistance was followed by a process of negotiated decision-making with family members and professionals, resulting in an experience by patients of ‘letting go’ when they reluctantly agreed to be admitted to hospital.^44^ Viewed retrospectively, participants often reported that if they had sought help earlier, then their condition may have been better managed or they may have avoided hospital admission. It was concluded that only in cases where self-care maintenance and monitoring could be performed effectively or ‘adequately’ that visits to hospital could be safely avoided.^54^

### Evidence on quality of life

The majority of studies reviewed which presented findings related to self-care and quality of life (QOL) found no significant differences between populations practising self-care when compared to usual care populations.^51,56,62,64^

In a cross-sectional study, higher self-care maintenance was associated with higher physical and emotional quality of life, and higher self-care management was associated with lower emotional quality of life.^42^ In contrast, two RCTs found non-significant results regarding self-care maintenance in cancer^51^ and self-care management in dementia.^62^

Studies on self-care monitoring produced either positive or non-significant quality of life results. In an RCT, a ‘Care Notebook’ was implemented as a self-monitoring QOL intervention for patients with cancer receiving palliative care^52^ and found a significant improvement in patients’ global QOL and physical function scores. However, an observational mixed-methods study did not find any significant differences between when comparing the same population pre- and post-intervention.^56^

### Evidence on cost

Evidence on the cost of self-care was limited with only three studies examining maintenance^45^ and monitoring.^49,65^ Two studies of cost-utility analyses of specific self-care interventions found them to be beneficial.^45,49^ The most methodologically robust was a cost-effectiveness study using a meta-analysis design; two self-care monitoring interventions in cancer (PainCheck and the Tackling Cancer Pain Toolkit) both demonstrated lower cost per quality-adjusted life-year than usual care.^49^ Cost was calculated from a proxy-completed questionnaire on utilisation of community services (GP visits and community nurse contacts) and secondary care services (hospital admissions, visits to Accident and Emergency Departments and hospice stays).

A specific self-care monitoring intervention for patients with incurable cancer, Oncokompas, did not impact costs and was seen to be slightly less effective compared to usual care.^65^ Regarding self-care maintenance, a feasibility mixed-methods cost–utility analysis on a small sample of 15 persons with cancer suggested that a self-management toolkit for end of life pain was cost-effective compared with standard care alone and could lead to cost savings.^45^

## Discussion

### Main findings

Studies centred mainly on either self-care monitoring or maintenance and underrepresented individual patient strategies to manage their conditions, which form a larger part of patients’ self-care activities.^18^ Across the studies reviewed, irrespective of diagnosis, self-care maintenance and monitoring could be viewed as an increasing burden for patients approaching the end of life. This is concerning given the potential for patients to continue self-care past the point it is feasible and perhaps becomes detrimental.

Research on care use was limited to acute care or hospitalisation at the end of life. We also found the current evidence base on how self-care affects quality and cost to focus mainly on the earlier stages of the end of life where patients were relatively able to self-care for their conditions. According to the studies reviewed, ensuring a smooth transition between self-care maintenance and formal care, in order to avoid sudden crises was often reported to be a considerable challenge. Qualitative data revealed that, towards the end of life, patients may often postpone seeking help until the point where they require emergency attention; at this point they may perceive that their own efforts to manage their disease have ‘failed’. Taken with studies examining participant perspectives on self-care, self-care towards the end of life inevitably reaches a point of ‘failure’. Our findings here resonate with others who note that towards the end of life, patients valued support that enabled them to maintain their independence and remain at home for as long as possible.^68^ When staying at home is a key patient priority, self-care can lead to crises requiring urgent hospital admission.

The evidence that did exist on care use was equivocal on the contributions of self-care maintenance and self-care management. Some studies suggested that self-care monitoring increased hospitalisations towards the end of life, which may be due to patients being more aware of their symptoms which would encourage them to seek help. Care towards the end of life is multi-faceted and may require a mixture of formal and informal care services, not limited to hospital acute care.

Our review showed that self-care interventions can be cost-effective towards the end of life, though findings are limited to small-scale feasibility studies and depend on how self-care is operationalised. The lack of uniformity in how quality of life was measured also limited comparison between studies with a potential negative impact of self-monitoring on quality of life being observed. A research gap that we highlight is that less reliance on formal care services implies a larger role of patient and family involvement including out-of-pocket payments for care; this is especially pertinent for patients approaching the end of life where costs become particularly high.^69^ Given the focus of the research base on service utilisation, quality and cost in formal settings, the scope for individuals to meet the personal costs associated with self-care is a particular concern, especially for individuals and families with lower socio-economic status. Future analyses on cost and cost-effectiveness should focus on taking self-financing of care and out-of-pocket expenditure into account.

Self-care at the end of life poses challenges to patients in how they provide their own care. Although our study found that self-care was viewed as burdensome by individuals, the burden of care can be felt unequally within specific patient populations. It has been noted that those with a lower socio-economic status who struggle to finance their own care, self- or otherwise, due to financial constraints and health literacy deficits.^70^

Burden is also felt unequally between different cultures and ethnicities.^71^ An important consideration to contextualise our findings is that some patient populations have more social resources to support a person’s individual care. As a result the burden for care may shift away from the individual to those around them with gender operating as important dynamic: female relatives are more likely to provide care for the patient.^66,72^ In the context of self-management support, informal carers take on roles that address deficits in the individual’s self-care abilities.^17^ For those lacking the informal caregiving capacity a reliance on, sometimes costly, professional support may be a further exacerbator of socioeconomic inequities.

More broadly, existing reviews on self-care outcomes such as healthcare use, quality and cost focus on the early stages of chronic disease and typically exclude populations at the end of life.^73–75^ Research about self-care in severe illness has produced inconclusive findings across multiple studies^76,77^ and is largely limited to the study of hospital admissions.^78^ Understanding how care needs can be met and financed in this population is a priority for future research on self-care towards the end of life.

### What this study adds?

Our study provides a new insight into how stakeholders at the end of life view the practice of self-care in its three facets of maintenance, management and monitoring. Uniquely, our review sheds new light on how self-care transitions from the advanced stages of disease towards the end of life with a focus on its impacts to utilisation, quality and cost across a variety of diagnoses. We distinguish our review from previous reviews in the literature that have centred on the experiences of persons with cancer^20–22^ and in acute settings or hospitalisations.

### Limitations of the study

This review sought to systematically identify and synthesise the published evidence on self-care towards and at the end of life. With the assistance of a professional medical librarian (IK), a comprehensive range of electronic databases were searched with additional journal hand searching and reference and citation searches located further relevant papers. Particular to our search strategy, we excluded conference abstracts, although efforts were made to locate any papers that were published based on the original conference abstracts through citation searching. Certain search terms such as ‘self-help’, ‘self-neglect’ and ‘assisted dying’ were excluded as they were deemed out of scope. We excluded any papers that focussed on remote digital health or virtual wards where there was not a significant component of self-care. The review was limited in defining the end-of-life stage for conditions when screening potentially relevant papers. The use of multiple reviewers (JG, JS, BA) helped to mitigate this but was still a potential issue with our overall review method. There were only three studies included from low- and middle-income countries (Uganda and Brazil; the findings of our review over-represent higher income countries.

## Conclusion

The research base on self-care in towards the end of life is limited and a growing field of inquiry. We found that for patients, self-care maintenance and monitoring could be experienced as an increasingly burdensome activity as illness progressed and their symptoms became more severe towards death. Self-care did initially increase patients’ sense of independence yet also came with a fear of mismanaging medication. Self-care monitoring could be particularly problematic as professionals explicitly mistrusted patient self-reported outcomes and felt that it could add to their workloads. Evidence also suggests that self-care monitoring could increase the number of hospitalisations in heart failure. Resisting hospital admission was a factor in delaying requests for service support. Patients more proficient at self-care maintenance are less likely to be hospitalised towards the end of life, though the overall quality of care is not clear from the current evidence with some studies suggesting negative effects. Research on cost-effectiveness suggests that self-care towards the end of life can be cost-effective although the research base is limited to hospital use, giving an incomplete picture of care use.
